# Adipose tissue dysregulation and reduced insulin sensitivity in non-obese individuals with enlarged abdominal adipose cells

**DOI:** 10.1186/1758-5996-4-42

**Published:** 2012-09-19

**Authors:** Ann Hammarstedt, Timothy E Graham, Barbara B Kahn

**Affiliations:** 1 The Lundberg Laboratory for Diabetes Research, Center of Excellence for Metabolic and Cardiovascular Research, Department of Molecular and Clinical Medicine, the Sahlgrenska Academy at the University of Gothenburg, Gothenburg, SE-413 45, Sweden; 2 Division of Endocrinology, Diabetes and Medicine, Department of Medicine, Beth Israel Deaconess Medical Center and Harvard Medical School, Boston, MA, USA

**Keywords:** Adipocyte cell size, BMI, Insulin sensitivity, GLUT4, Adiponectin, RBP4

## Abstract

**Background:**

Obesity contributes to Type 2 diabetes by promoting systemic insulin resistance. Obesity causes features of metabolic dysfunction in the adipose tissue that may contribute to later impairments of insulin action in skeletal muscle and liver; these include reduced insulin-stimulated glucose transport, reduced expression of GLUT4, altered expression of adipokines, and adipocyte hypertrophy. Animal studies have shown that expansion of adipose tissue alone is not sufficient to cause systemic insulin resistance in the absence of adipose tissue metabolic dysfunction. To determine if this holds true for humans, we studied the relationship between insulin resistance and markers of adipose tissue dysfunction in non-obese individuals.

**Method:**

32 non-obese first-degree relatives of Type 2 diabetic patients were recruited. Glucose tolerance was determined by an oral glucose tolerance test and insulin sensitivity was measured with the hyperinsulinaemic-euglycaemic clamp. Blood samples were collected and subcutaneous abdominal adipose tissue biopsies obtained for gene/protein expression and adipocyte cell size measurements.

**Results:**

Our findings show that also in non-obese individuals low insulin sensitivity is associated with signs of adipose tissue metabolic dysfunction characterized by low expression of GLUT4, altered adipokine profile and enlarged adipocyte cell size. In this group, insulin sensitivity is positively correlated to GLUT4 mRNA (R = 0.49, p = 0.011) and protein (R = 0.51, p = 0.004) expression, as well as with circulating adiponectin levels (R = 0.46, 0 = 0.009). In addition, insulin sensitivity is inversely correlated to circulating RBP4 (R = −0.61, 0 = 0.003) and adipocyte cell size (R = −0.40, p = 0.022). Furthermore, these features are inter-correlated and also associated with other clinical features of the metabolic syndrome in the absence of obesity. No association could be found between the hypertrophy-associated adipocyte dysregulation and HIF-1alpha in this group of non-obese individuals.

**Conclusions:**

In conclusion, these findings support the concept that it is not obesity *per se,* but rather metabolic dysfunction of adipose tissue that is associated with systemic insulin resistance and the metabolic syndrome.

## Introduction

There is currently a global epidemic of Type 2 diabetes due to recent changes in lifestyle. Obesity is the major factor promoting the diabetes epidemic and this suggests that the expanded adipose tissue is a major driver through induction of obesity-associated insulin resistance [1]. In agreement with this concept, insulin resistance is observed locally in the adipose tissue long before glucose intolerance develops [2]. Early cellular markers of insulin resistance in adipose tissue are reduced adipose cell GLUT 4 and IRS 1 protein expression [3-6]. Interestingly, this is seen around four times more frequently in individuals with a genetic predisposition for type 2 diabetes than in subjects lacking a genetic predisposition [5]. The reason for this is currently unclear but the phenomenon suggests an association between genetic predisposition for type 2 diabetes and a dysregulated adipose tissue.

We have recently shown that the ability to differentiate preadipocytes into adipocytes is reduced in cells from adipose tissue characterized by enlarged fat cells [7]. This seems to predominantly be due to impaired preadipocyte differentiation rather than a lack of early precursor cells including mesenchymal stem cells [7]. These results clearly indicate that adipose tissue dysfunction is related to adipose cell enlargement. Experiments in animal models also support this concept since, for instance, overexpressing adiponectin in adipose tissue leads to a marked hypercellular obesity without adipose cell enlargement and the animals are at least as insulin sensitive as the lean wildtype mice [8]. In addition, over expression of GLUT4 in adipocytes leads to hyperplastic obesity and enhanced glucose tolerance [9]. The increased GLUT4 in fat even overcomes insulin resistance in muscle resulting from genetic deletion of GLUT4 in muscle [10]. Clearly, adipose tissue function is important for whole body glucose homeostasis.

In this study we examined if adipose tissue dysfunction is more closely related to adipocyte hypertrophy rather than to BMI in man. We investigated GLUT4 expression in adipose cells as a marker of adipose tissue dysregulation in relation to whole-body insulin sensitivity, serum levels of adiponectin and RBP4, as well as the relationship to adipose cell size in a population of non-obese subjects.

## Material and methods

### Subjects

All subjects included in the study were healthy non-diabetic offspring of parents with type 2 diabetes. Clinical and biochemical characteristics of the study population are shown in Table 1. The study was approved by the ethical committee of the University of Gothenburg and performed in accordance with the Declaration of Helsinki. Written consent was obtained from each subject.

**Table 1:** Clinical characteristics of the studied individuals

**Variable**	**Mean ± SD**
Sex (male/female)	10/22
Age (years)	42 ± 6
Height (m)	1.73 ± 0.08
Weight (kg)	75.7 ± 10.3
BMI (kg/m2)	25.2 ± 2.4
Waist (cm)	87 ± 8
Hip (cm)	104 ± 5
WHR	0.83 ± 0.08
Glucose (mmol)	4.9 ± 0.5
Insulin (mU/L)	7.1 ± 3.1
GIR (mg/min/kgLBM)	12.6 ± 3.9
OGTT 2 h glucose (mmol/l)	6.5 ± 1.7
HOMA-index	1.68 ± 0.84
HbA1c (%)	4.14 ± 0.26
s-triglycerides (mmol/l)	1.08 ± 0.65
s-HDL cholesterol (mmol/l)	1.51 ± 0.46
s-LDL cholesterol (mmol/l)	2.86 ± 0.89
Blood pressure syst (mmHg)	116 ± 9
Blood pressure diast (mmHg)	73 ± 7
s-Adiponectin (ug/ml)	10.8 ± 4.3
s-RBP4 (RQ)	1.40 ± 0.38
Adipocyte cell size (ug)	0.47 ± 0.17

*GIR* Glucose infusion rate during the euglycemic clamp.

*OGTT* oral glucose tolerance test.

*WHR* waist/hip ratio.

### Biochemical and anthropometric measures

Height and weight were measured to the nearest cm and 0.1 kg and BMI calculated as kg body weight divided by height (m) squared. Fasting blood samples were drawn after an over night fast followed by an OGTT (75 g glucose) to evaluate glucose tolerance (blood samples were taken at 0, 30, 90 and 120 min). Circulating plasma glucose was determined using a photometric method by the accredited central hospital laboratory and insulin concentrations by a micro-particle enzyme immunoassay (Abbott Laboratories, Tokyo, Japan). At 60 min after a glucose bolus a hyperinsulinaemic-euglycaemic clamp was initiated and carried out for the next 120 min (insulin infusion 40 mU, m^-2^, min^-2^) to evaluate insulin sensitivity. Blood glucose was clamped at 5 mmol/l by infusion of 20% glucose at various rates according to the blood glucose measurements performed at 5 min intervals. The mean amount of glucose infused during the last hour was used to calculate the rate of whole-body glucose uptake. Non-esterified fatty acids in serum were measured by an enzymatic colorimetric method (Wako Chemicals, Neuss, Germany) while other plasma lipid concentrations were determined with an automated Cobra Mira analyser (Hoffman-LaRoche, Basel, Switzerland). Circulating adiponectin levels were measured in serum by a human adiponectin ELISA-kit (B-Bridge International, Sunnyvale, CA, USA) according to the manufacturers instructions and serum RBP4 by quantitative Western Blot [11]. Information of physical activity was collected by a questioner and expressed as number of times per week of exercise at least 20 min.

### Adipocyte isolation

Human abdominal subcutaneous adipose tissue was obtained in the fasting state by needle biopsy. Isolation of adipocytes was performed essentially as previously described [7]. Briefly, biopsies were washed to remove traces of blood and treated with collagenase (1 mg/ml) (Sigma, St Louise, MO, USA) for ~60 min at 37°C in a shaking waterbath. Isolated adipocytes were filtered through a 250 μm nylon mesh, washed with fresh medium. Adipocyte cells were placed on a siliconized glass slide and 100 consecutive cell diameters were measured with a calibrated ocular.

### Cell lysate and Western blot

Isolated human adipocytes were separated from medium by centrifugation through dinonyl phthalate. Lysis buffer was added, samples briefly vortexed and rocked for 2 h at 4°C. Insoluble material was sedimented by centrifugation and supernatant collected and stored at −80°C prior to use [12]. Protein concentration was measured using the bicinchonic acid method (Pierce, Rockford, IL, USA). Protein were separated on SDS-PAGE as described [4] and immunoblotted with an anti-GLUT4 antibody (Chemicon, Temecula, CA, USA).

### RNA extraction and quantification

Total cellular RNA was extracted from abdominal subcutaneous adipose tissue biopsies with the guanidinium thiocyanate method as described [13]. Gene expression was analyzed with the ABI PRISM 7900HT sequence detection system (TaqMan, Applied biosystems, Foster City, CA, USA). Gene-specific primers and probes were designed using the Primer Express software (Applied biosystems, Foster City, CA, USA) GLUT4: Fp TCTGGCATCAATGCTGTTTTCTAT, Rp ACCAACAACACCGAGACCAAG, probe TGACCACACCAGCTCCTATGGTGGC; C/EBPalpha: Fp CCAAGAAGTCGGTGGACAAGA, Rp GCGCACCGCGATGTTGTT, probe CGCCGCACCCGGTACTCGTT; HIF-1alpha: Fp AAATACATGGGATTAACTCAGTTTGAA, Rp GGCCATTTCTGTGTGTAAGCAT, probe CATCCATGTGACCATGAGGAAATGAGAGA; VEGF: Hs00173626_m1 (Applied Biosystems, Foster City, CA, USA). Each sample was run in duplicate and the quantity of a particular gene in each sample was normalized to ribosomal *18 s* RNA.

### Statistical analysis

All data are presented as mean ± SD. Data was tested for normality and, if appropriate, Log transformed. Linear correlations and adjustment for gender and exercise were performed using PASWstatistics (SPSS Inc). P-value <0.05 was considered to be significant. P-values were adjusted for multiple testing using the Bonferroni-Holm correction algorithm (SAS).

## Results

We characterized adipose tissue GLUT 4 protein and gene expression in 32 individuals with BMI range 20.8-29.7 and a genetic predisposition for type 2 diabetes (first degree relatives; FDR). These individuals were part of a large inter-European study, EUGENE2, relating phenotype to genotype. The inclusion criteria and phenotyping procedures have been reported previously [14].

The clinical characteristics of the cohort studied here are shown in Table 1.

### Body composition in relation to insulin sensitivity

Insulin sensitivity measured with the euglycemic clamp technique was significantly negatively correlated with adipose cell size, as expected [15] as well as with waist/hip ratio while the correlation to BMI was not significant in these non-obese individuals (Table 2). Furthermore, insulin sensitivity correlated with, s-HDL-cholesterol, total s-adiponectin levels, and adipose tissue GLUT 4 protein expression and mRNA levels, and inversely with s-triglycerides and s-RBP4 (Table 2). Adjusting for gender did not significantly alter the results except for s–triglycerides that were no longer significant after adjustment (p = 0.056), also after adjustment for gender and exercise s-HDL was no longer significantly correlated with insulin sensitivity (p = 0.059). Adjusting for exercise alone did not affect the results. These results support the concept that insulin sensitivity is more closely related to adipose cell size and adipose tissue distribution than to BMI.

**Table 2:** Correlation between insulin sensitivity and adipose cell size with phenotype

	**Insulin sensitivity**						**Adipocyte cell size**					
	**All**		**Men**		**Women**		**All**		**Men**		**Women**	
	***R-value***	***P-value (P***_***corr***_***)***	***R-value***	***P-value (P***_***corr***_***)***	***R-value***	***P-value (P***_***corr***_***)***	***R-value***	***P-value (P***_***corr***_***)***	***R-value***	***P-value (P***_***corr***_***)***	***R-value***	***P-value (P***_***corr***_***)***
BMI	−0.05	0.79 (0.98)	−0.06	0.88 (1)	0.07	0.77 (1)	0.53	0.002 (0.022)	0.70	0.025 (0.25)	0.43	0.046 (0.30)
WHR	−0.49	0.004 (0.044)	−0.36	0.31 (1)	−0.49	0.02 (0.22)	0.36	0.041 (0.25)	0.54	0.11 (0.77)	0.47	0.026 (0.26)
Glucose	−0.13	0.49 (0.98)	−0.55	0.10 (1)	0.16	0.47 (1)	0.30	0.092 (0.28)	0.48	0.16 (0.84)	0.21	0.36 (0.72)
Insulin	−0.34	0.055 (0.22)	−0.66	0.037 (0.44)	−0.21	0.36 (1)	0.52	0.002 (0.022)	0.64	0.045 (0.41)	0.49	0.028 (0.26)
HbA1c	−0.24	0.18 (0.18)	−0.44	0.21 (1)	−0.11	0.63 (1)	0.28	0.13 (0.28)	0.43	0.21 (0.84)	0.21	0.34 (0.72)
GIR	–	–	–	–	-	-	−0.40	0.022 (0.18)	−0.40	0.26 (0.84)	−0.44	0.043 (0.30)
s-triglycerides	−0.39	0.028 (0.14)	−0.39	0.26 (1)	−0.36	0.10 (0.50)	0.51	0.003 (0.027)	0.73	0.018 (0.22)	0.41	0.061 (0.30)
s-HDL	0.43	0.019 (0.13)	−0.04	0.92 (1)	0.57	0.009 (0.11)	−0.31	0.10 (0.28)	0.14	0.71 (0.84)	−0.59	0.006 (0.072)
s-Adiponectin	0.46	0.009 (0.081)	0.37	0.29 (1)	0.42	0.05 (0.31)	−0.54	0.001 (0.012)	−0.71	0.022 (0.24)	−0.55	0.008 (0.088)
s-RBP4	−0.61	0.003 (0.036)	−0.43	0.26 (1)	−0.59	0.034 (0.31)	0.41	0.059 (0.26)	0.53	0.14 (0.84)	0.40	0.18 (0.72)
GLUT4 mRNA	0.49	0.011 (0.088)	0.18	0.62 (1)	0.49	0.02 (0.22)	−0.40	0.023 (0.18)	−0.44	0.21 (0.84)	−0.45	0.034 (0.27)
GLUT4 protein	0.51	0.004 (0.044)	0.57	0.11 (1)	0.45	0.034 (0.31)	−0.35	0.051 (0.26	−0.59	0.095 (0.76)	−0.27	0.23 (0.72)
Adipose cell size	−0.40	0.022 (0.13)	−0.40	0.26 (1)	−0.44	0.043 (0.31)	–	–	–	–	–	–

*GIR* Glucose infusion rate during the euglycemic clamp.

*WHR* waist/hip ratio.

P_corr_: P-value Bonferroni-Holm corrected.

### Abdominal adipose cell size and markers of insulin sensitivity

**Figure 1:**
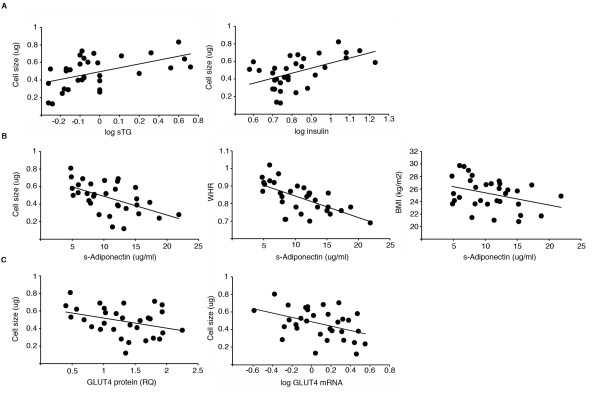
**Adipocyte cell size correlations.** (**A**) Adipocyte cell size is positively correlated to circulating triglycerides (R = 0.51, p = 0.003) and circulating insulin levels (R = 0.52, p = 0.002). (**B**) Adipose cell size (R = −0.54, p = 0.001), WHR (R = −0.63, p < 0.001) and BMI (R = −0.35, p = 0.05) are all significantly and inversely associated with circulating adiponectin levels. (**C**) Adipose cell size tends to be negatively associated with the expression the glucose transporter GLUT4 protein (R = −0.35, p = 0.051) and is negatively associated with mRNA (R = −0.40, p = 0.023) in the adipose tissue in non-obese subjects.

Adipose cell size also correlated with other known metabolic consequences of insulin resistance including circulating insulin levels and total triglyceride levels (Table 2 and Figure 1a).

More importantly, adipose cell size, like waist/hip ratio, correlated negatively with circulating adiponectin levels. BMI also tended to correlate with circulating adiponectin levels. The correlation was, however, not as strong as with adipocyte cell size (Figure 1b). Furthermore, adipose cell size was inversely correlated to adipose tissue *GLUT4* gene expression and a trend for this was also found at protein level (Figure 1c). In addition, a borderline significant negative correlation was found with circulating RBP4 levels (Table 2) while no association could be found between circulating RBP4 and BMI.

Adjusting for gender resulted in a statistical significant correlations between cell size and GLUT4 protein (p = 0.033) and also negatively with RBP4 (p = 0.046) and did not significantly affect other associations. Adjusting for exercise did not change the results.

### Adipose tissue GLUT 4 and circulating levels of insulin sensitivity or resistance markers

**Figure 2:**
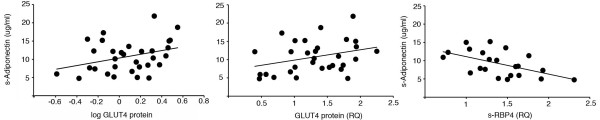
**Circulating factors.** (**A**) Circulating total adiponectin level tends to correlate with GLUT4 mRNA (R = 0.34, p = 0.059) and protein (R = 0.32, p = 0.08) expression in the adipose tissue and is negatively correlated with circulating serum RBP4 levels (R = −0.54, p = 0.009).

We then examined GLUT4 expression in the adipose tissue in relation to insulin sensitivity and circulating adiponectin and RBP4 levels. Circulating adiponectin levels tended to correlate positively with GLUT4 gene and protein expression. Adjusting for exercise did, however, result in a significant correlation between adiponectin and GLUT4 mRNA (p = 0.041) whereas adjusting for gender made the associations non-significant. Adiponectin correlated negatively with serum RBP4 levels (Figure 2), which was not affected by exercise. We also examined the expression of *C/EBPalpha*, an important transcription factor for adipocyte insulin sensitivity [16], and found, as expected, a significant and positive correlation with both GLUT4 protein and gene expression (data not shown). Adiponectin levels also correlated positively with degree of insulin sensitivity (Table 2), HDL-cholesterol and negatively with fasting insulin and HbA1c levels (Table 3). Thus, both markers of insulin sensitivity; i.e.; GLUT4 expression in the adipose tissue and serum adiponectin, which is only expressed in and secreted by the adipose cells, are closely correlated to each other and show a similar profile.

**Table 3:** Correlation between phenotype and circulating adiponectin levels

	**Adiponectin**					
	**All**		**Men**		**Women**	
	***R-value***	***P-value (P***_***corr***_***)***	***R-value***	***P-value (P***_***corr***_***)***	***R-value***	***P-value (P***_***corr***_***)***
BMI	−0.35	0.05 (0.20)	−0.69	0.028 (0.25)	−0.18	0.42 (1)
WHR	−0.63	<0.001 (0.01)	−0.67	0.034 (0.27)	−0.52	0.014 (0.14)
Glucose	−0.35	0.052 (0.20)	−0.49	0.15 (0.60)	0.05	0.84 (1)
Insulin	−0.43	0.013 (0.091)	−0.64	0.047 (0.31)	−0.34	0.12 (0.96)
HbA1c	−0.39	0.03 (0.18)	−0.35	0.32 (0.96)	−0.27	0.22 (1)
s-triglycerides	−0.38	0.032 (0.18)	−0.64	0.044 (0.31)	−0.15	0.49 (1)
s-HDL	0.50	0.005 (0.045)	0.27	0.45 (0.96)	0.52	0.019 (0.17)
s-RBP4	−0.54	0.009 (0.072)	0.62	0.08 (0.40)	0.43	0.14 (0.98)
GLUT4 mRNA	0.34	0.059 (0.20)	0.79	0.006 (0.06)	0.18	0.43 (1)
GLUT4 protein	0.32	0.083 (0.20)	0.34	0.37 (0.96)	0.16	0.49 (1)

*WHR* waist/hip ratio.

P_corr_ P-value Bonferroni-Holm corrected.

### Adipose tissue dysregulation, inflammation and hypoxia

Adipose tissue inflammation is increased in hypertrophic obesity and promotes dysregulated adipose tissue biology [17]. In line with these findings, we have previously shown that the inflammatory cytokine IL-6 is elevated in hypertropic obesity and that the interstitial concentration correlates with cell size [18]. Another possible candidate for adipose tissue dysregulation associated with hypertrophic obesity is HIF-1alpha since cellular hypoxia has been demonstrated to occur when the adipose cells expands [19]. However, we found no correlation between either adipose cell size, insulin sensitivity or any of the markers of insulin resistance with *HIF-1alpha* mRNA levels or the HIF-1alpha-induced gene *VEGF* in these non obese subjects (Table 4). Adjusting for gender and/or exercise did not affect the results. The results from these analyses may have been affected by the reduced number of subjects included due to limited mRNA or tissue availability.

**Table 4:** Correlation between phenotype and HIF-1alpha and VEGF expression

	**HIF-1alpha**		**VEGF**	
	***R-value***	***P-value (P***_***corr***_***)***	***R-value***	***P-value (P***_***corr***_***)***
BMI	−0.15	0.54 (1)	−0.14	0.62 (1)
WHR	0.03	0.89 (1)	−0.56	0.032 (0.42)
Glucose	0.13	0.61 (1)	−0.24	0.39 (1)
Insulin	0.07	0.76 (1)	−0.16	0.57 (1)
HbA1c	0.15	0.55 (1)	−0.10	0.73 (1)
GIR	−0.17	0.48 (1)	0.23	0.42 (1)
s-triglycerides	−0.06	0.80 (1)	−0.44	0.10 (1)
s-HDL	−0.06	0.82 (1)	0.22	0.46 (1)
s-Adiponectin	−0.08	0.76 (1)	0.43	0.11 (1)
s-RBP4	0.28	0.26 (1)	−0.07	0.81 (1)
GLUT4 mRNA	0.08	0.74 (1)	0.54	0.039 (0.47)
GLUT4 protein	−0.23	0.36 (1)	0.43	0.12 (1)
Adipose cell size	0.06	0.80 (1)	−0.04	0.89 (1)

*GIR* Glucose infusion rate during the euglycemic clamp.

*WHR* waist/hip ratio.

P_corr_: P-value Bonferroni-Holm corrected.

## Discussion

A number of studies have established that adipose tissue dysfunction contributes to metabolic dysfunction and type 2 diabetes. Enlargement of adipocyte cell size has been shown to be associated with adipose tissue dysfunction and, in addition, to predict later development of type 2 diabetes in Pima Indians, a population with high propensity for obesity and type 2 diabetes [20], as well as in a Swedish cohort of middle-aged women [21]. Although obesity is a major risk factor for the development of type 2 diabetes, not all obese individuals become insulin resistant or develop type 2 diabetes. Recently, it was shown by Klöting et al. [22] that insulin-sensitive, severely obese individuals have smaller adipocyte size compared to an equally obese group matched for age, sex and body fat. This finding was also associated with reduced tissue inflammation and higher insulin-stimulated glucose uptake at least in omental adipose tissue. Furthermore, by comparing non-obese subjects with a known genetic predisposition for either type 2 diabetes or obesity, we have recently shown that for a given amount of body fat individuals with a genetic predisposition for type 2 diabetes had an inappropriate enlargement of their abdominal adipocyte cell size. This difference was evident when they were compared to subjects with a genetic predisposition for obesity or to control subjects lacking a known genetic predisposition [23]. These findings indicate that adipocyte hypertrophy combined with an “obese phenotype” is present in the abdominal adipose tissue long before type 2 diabetes develops and that this is related to insulin resistance rather than obesity *per se*. Clearly, adipocyte cell size and function are related to whole body insulin sensitivity.

The cohort studied in this report was part of the inter-European EUGENE 2 program ( http://www.eugene2.com) [14]. This program focused on carefully phenotyping individuals at risk for type 2 diabetes by virtue of having at least one first degree relative with this condition. This cohort has undergone several genotyping studies and is followed prospectively in order to identify future diabetes development. The individuals included in this study were healthy and non-obese, but they are a high-risk group even though current obesity was not part of the risk profile. Furthermore, they are more insulin-resistant, as a group, than matched control subjects without a family history of type 2 diabetes [5].

The results from the present study show that markers of adipose tissue dysregulation are present already in these otherwise healthy individuals. Adipose cell size, GLUT 4 protein and mRNA expression as well as circulating levels of adiponectin and RBP4 were all markers for degree of insulin sensitivity. Interestingly, adipose cell size was positively correlated with serum RBP4, which is consistent with previous findings [24], and inversely with adiponectin levels as well as with *GLUT 4* expression. These findings are consistent with the concept that adipose cell expansion, even over the limited range of BMI in this cohort, is associated with insulin resistance as well as markers of a dysregulated adipose tissue measured as low GLUT4 expression and circulating levels of adiponectin and high serum RBP4 levels. The associations also suggest that these molecules may have common gene regulatory sites such as C/EBPalpha although no correlation was found between mRNA levels for this transcription factor and adiponectin or RBP4 levels. It should also be pointed out that we did not measure *APM1* mRNA levels since this molecule is subject to important post-transcriptional modifications as well as a regulated secretion pathway [25], thus making mRNA levels less dependent than total protein secreted and present in the blood. Virtually all clinical studies on the role of adiponectin have also focused on the circulating levels of this protein. It is also noteworthy that while circulating adiponectin levels were only border-line correlated to BMI and serum RBP4 levels not at all, the association with adipose cell size was highly significant. These findings are in line with the results presented by Klöting et al. where circulating adiponectin is decreased and RBP4 increased in equally obese individuals with enlarged adipocytes and reduced insulin sensitivity [26].

Interestingly, insulin sensitivity was most closely related to waist/hip ratio and adipose cell size while BMI was a poor marker. The lack of correlation with BMI is probably due to the relatively limited range of BMI in this cohort since BMI is well known to be associated with insulin sensitivity in large population samples with different degree of obesity. Thus, this study shows that adipose cell size and adipose tissue distribution are more sensitive parameters over a relatively limited range of BMI in a cohort of non-obese subjects. In addition, these results show that adipose tissue dysregulation does not require obesity per se but rather hypertrophic adipose cells. The hypertrophic characteristics of the adipose tissue is probably due to inability to recruit and/or differentiate preadipocytes which results in excessive lipid deposition in, and enlargement of, pre-existing adipocytes. We recently found that even though precursor cells are available in adipose tissue there seems to be a blockage of the commitment and/or initiation of adipocyte differentiation leading to impaired preadipocyte recruitment in individuals with enlarged fat cells [7]. This is in line with recent findings showing that the number of new adipocytes generated each year is reduced in subjects with adipocyte hypertrophy while the relative death rate is unchanged [27]. What constitutes the blockage is still unknown, but clearly the resulting adipocyte hypertrophy is associated with adipose tissue dysfunction. We here show that this is also related to reduced insulin sensitivity combined with a metabolic risk profile and markers of adipose tissue dysregulation. It is well known that adipose tissue distribution differs between men and women but, interestingly, it has also been shown that the proportion of early-differentiated adipocytes, measured as percentage of PPARgamma expressing cells in the subcutaneous adipose tissue is increased in women when compared to men indicating that there may be important gender-related differences in pre-adipocyte recruitment, proliferation and differentiation potential [28]. Our results show that the correlation between adipocyte cell size and GLUT4 as well as the insulin resistance marker RBP4 are affected by gender. This is also true for the association between adiponectin and GLUT4, both markers of late adipocyte differentiation and function. These findings add further strength to the concept of gender differences in adipocyte differentiation and function. Tchoukalova et al. speculate that a possible mechanism could include gender-specific differences in the micro-environment and/or effects of sex-steroid hormones. Sex steroid-hormones have been shown to influence fat distribution [29] as well as adipocyte differentiation [30] and could well be responsible for the gender differences observed. Unfortunately, we did not measure sex-steroids in the present study. However, a recent paper investigating clinical characteristics associated with insulin sensitivity in women with polycystic ovary syndrome (PCOS) showed that the strongest predictors of insulin sensitivity in this group were adipocyte cell size, adiponectin and WHR, while sex steroid-hormones were excluded from the regression model [31]. Further studies are required to elucidate the importance of, and mechanisms behind, these gender-associated differences.

It is well established that enlarged adipose cells leads to infiltration of macrophages and other inflammatory cells, including mast cells [32-34]. The presence of inflammatory cells in the adipose tissue affects the micro-environment and can impair adipocyte differentiation [7,17]. Indeed, macrophage infiltration in the omental adipose tissue depot, together with circulating adiponectin was found to almost completely explain the degree of insulin sensitivity in severely obese individuals [26]. We previously measured the inflammatory cytokine IL6 in adipose tissue and showed that expression, secretion and, as a consequence, also interstitial levels of this cytokine were increased in the adipose tissue characterized by enlarged fat cells [18]. Thus, inflammation seems to follow adipose cell size enlargement and this is also associated with impaired adipocyte differentiation [35]. Cellular hypoxia has also been implicated in adipose tissue dysregulation in obesity [19]. However, we found no relationship between *HIF-1alpha* mRNA levels, or *VEGF*, which is an HIF-1alpha-regulated gene [36], and adipose cell size or any marker of insulin resistance. This is in agreement with previously reports in obese individuals where the expression in subcutaneous adipose tissue was unrelated to the degree of insulin sensitivity or cell size. In contrast, the expression of HIF-1alpha has been shown to be up-regulated in insulin resistant omental adipose tissue in severe obese individuals [26]. Thus, although HIF-1alpha may play a role in severe obesity, we did not find any association between insulin sensitivity and HIF-1alpha in this small group of individuals with hypertrophic adipocytes.

The results of the present study clearly show that enlarged abdominal adipose cells are associated with reduced systemic insulin sensitivity irrespective of whether obesity is present or not.

A likely reason for the insulin resistance is altered RBP4 and adiponectin levels as well as an inability to store additional lipids in the subcutaneous depot during weight gain. This leads to storage in ectopic sites including visceral depots, liver and muscle which, in turn, further promotes insulin resistance (Reviewed in [15,37]). Elegant experiments in mouse models have indeed shown that mice overexpressing adiponectin in the subcutaneous adipose tissue become grossly obese with hypercellular adiposity as a consequence of new preadipocyte recruitment and differentiation. The changes associated with this transgene did not impair insulin sensitivity at all [8]. The present results further support the concept that pre-adipocyte recruitment and hypercellular obesity can prevent the development of insulin resistance.

The present study is limited by its small number of subjects and using the quite conservative methods available to correct for multiple testing leaves few significant correlations. However, regardless of these limitations, the results provide important information in a high-risk cohort of first-degree relatives to type 2 diabetic patients showing that a dysregulated adipose tissue occurs early and is associated with insulin resistance. Future studies, such as long-term follow-up studies of the EUGENE 2 cohorts may provide further evidence for this concept as a risk to develop type 2 diabetes as well.

## Conclusion

The findings in the present paper support the concept that it is not obesity *per se,* but rather metabolic dysfunction in the adipose tissue that is associated with systemic insulin resistance and the metabolic syndrome. Future prospective studies may provide final evidence of this concept and the relative importance of its different components.

## Abbreviations

GLUT4: Glucose transporter 4; IRS-1: Insulin receptor substrate-1; RBP4: Retinol-binding protein 4; BMI: Body mass index; OGTT: Oral glucose tolerance test; HDL: High-density lipoprotein; C/EBP: CCAAT/enhancer binding protein; HIF-1: Hypoxia induced factor-1.

## Competing interest

The authors declare that they have no competing interest**.**

## Authors’ contribution

AH and BBK participated in the planning and designing of the study and writing the manuscript. AH prepared the clinical samples and performed the analyses of the adipose tissue biopsies. TEG prepared samples and performed the RBP4 analysis and participated in writing the manuscript. All authors have read and approved the final manuscript.
